# The effects of tramadol on postoperative shivering after sevoflurane and remifentanil anesthesia

**DOI:** 10.1186/s12871-016-0295-x

**Published:** 2017-01-03

**Authors:** Taku Nakagawa, Miki Hashimoto, Yasunori Hashimoto, Kazuhiro Shirozu, Sumio Hoka

**Affiliations:** 1 Department of Anesthesiology, Hachinohe city Hospital, Aomori, Japan; 2 Department of Anesthesiology and Critical Care Medicine, Kyushu University Hospital, Fukuoka, Japan; 3 Operating Rooms, Kyushu University Hospital, 3-1-1 Maidashi, Higashi-ku, Fukuoka, 812-8582 Japan; 4 Department of Anesthesiology and Critical Care Medicine, Kyushu University Graduate School of Medical Sciences, Fukuoka, Japan

**Keywords:** Tramadol, Shivering, Remifentanil

## Abstract

**Backgrounds:**

Remifentanil has been reported to cause post-anesthetic shivering (PAS). Higher doses of remifentanil reportedly induce more intense PAS. Tramadol, a synthetic opioid that acts at multiple sites, is considered to be an effective treatment for PAS, but the evidence for its therapeutic benefit after remifentanil anesthesia is limited. We investigated the effect of tramadol on the incidence of PAS after remifentanil anesthesia.

**Methods:**

Sixty-three patients who had undergone upper abdominal surgery under general anesthesia were studied retrospectively. Tramadol was administered at induction of anesthesia. The patients were divided into four groups: HT(+), high dose remifentanil (1–1.5 μg/kg/min) with tramadol; HT(−), high dose remifentanil without tramadol; LT(+), low dose remifentanil (0.15–0.25 μg/kg/min) with tramadol; and LT(−), low dose remifentanil without tramadol. We recorded perioperative changes in nasopharyngeal temperature and episodes of PAS on emergence from anesthesia.

**Results:**

The incidences of PAS in both tramadol treatment groups were significantly lower than the groups that did not receive tramadol. Nasopharyngeal temperature after surgery fell significantly more from baseline in the tramadol treatment groups compared with the non-treatment groups.

**Conclusion:**

Tramadol administered at induction of anesthesia appears to suppress PAS following remifentanil anesthesia.

## Background

Remifentanil is an ultra-short acting opioid that contributes to rapid recovery after anesthesia [1]. It has, however, been reported that remifentanil increases the risk of post-anesthetic shivering (PAS) after abrupt withdrawal [2–6]. Moreover, it has also been reported that withdrawal from a high dose of remifentanil induces more intense PAS compared with a low dose [7]. Tramadol, a synthetic opioid that acts at multiple sites, is often used to treat PAS [8–16]; however, the evidence for its therapeutic benefit after remifentanil anesthesia is limited. We investigated the effect of tramadol on the incidence of PAS after high-dose (1–1.5 μg/kg/min) or low-dose (0.15–0.25 μg/kg/min) remifentanil anesthesia.

## Methods

Conduct of the study was approved by our institutional ethics committee (Hachinohe City Hospital, Hachinohe, Aomori, Japan) and registered at UMIN-CTR (UMIN000019785). The records of 63 patients who were all the patients undergoing upper abdominal surgery (not included laparoscopic surgery) under general anesthesia used sevoflurane and remifentanil for the maintenance between March 2007 and February 2012 were examined retrospectively. This study was observational, non-randomized and not controlled. The American Society of Anesthesiologists physical status of all patients was class 1 or 2.

Anesthesia was induced with midazolam (0.04 mg/kg) and remifentanil, and maintained with sevoflurane (1–1.5%) and remifentanil. Tramadol (3.5 mg/kg) was administered intravenously at the induction of anesthesia and an additional 1.5 mg/kg was given every 4 h thereafter. Patients were divided into four groups on the basis of remifentanil dose and tramadol administration: group HT(+) received high-dose remifentanil (1–1.5 μg/kg/min) with tramadol; group HT(−) received high-dose remifentanil without tramadol; group LT(+) received low-dose remifentanil (0.15–0.25 μg/kg/min) with tramadol; and group LT(−) received low-dose remifentanil without tramadol. The high-dose remifentanil group (1–1.5 μg/kg/min) was defined from the dose we have usually administered in upper abdominal surgery. The low-dose remifentanil group (0.15–0.25 μg/kg/min) was defined from the empirical dose we thought to be the minimum in upper abdominal surgery. We used Ultiva® (remifentanil hydrochloride) as remifentanil preparation. Intravenous tramadol was adopted for post operating shivering in our hospital in 2010. In this study, group LT(+) and group HT(+) were after 2011. Fentanyl, flurbiprofen (2 mg/kg, except when it is contraindicated as asthma, intestinal ulcer and reduced renal function) and intravenous patient controlled analgesia (IV-PCA) with fentanyl, or epidural anesthesia were used as postoperative analgesia. Flurbiprofen was administered 30 min before completion of surgery. Premedication was not administered. All fluids were pre-warmed. Episodes of PAS were identified from anesthetic and medical records. In our hospital, we have to record the occurrence of PAS as complication if shivering occurred. Then we could identify patients with shivering from past electronic chart. Post-anesthetic shivering was evaluated by not the same person, but each anesthesiologist was in charge. The evaluation was performed within one hour after surgery. Duration of surgery and anesthesia, and administration of flurbiprofen were also recorded. Core temperature was measured in the nasopharynx. A thermometer catheter (Temperature probe, Drager, USA) was placed immediately after induction of anesthesia and removed at the end of surgery by attending anesthesiologist; the difference between the two was calculated. In the period from 2007 to 2012, we did not apply active warming for surgical patients during anesthesia due to lack of the equipment. Alternatively to prevent body temperature decrease, room temperature of our operating room was maintained approximately at 25 °C as possible and an underbody warming blanket as well as a transfusion warming device were commonly used. These efforts might not remarkably reduce the mean nasopharyngeal temperature of patients during surgery.

### Statistical analysis

Based on a preliminary study, power analysis (α = 0.05, β = 0.20) indicated that fewer than 10 subjects per group would be needed to detect a significant difference in the incidence of PAS in the high- or low-dose remifentanil groups. Then fifteen subjects were needed per group, to account for potential protocol failures or dropouts. Data are presented as median [minimum, maximum]; n denotes the number of patients. Data were analyzed using the Prism 6 software package (GraphPad software, CA USA). Fisher’ exact test and the ordinary one- or two-way analysis of variance (ANOVA) were used for statistical analyses. Holm-Sidak test, Dunn’s or Tukey’s multiple comparison *post hoc* tests were performed for Fisher’s exact test, one-way one- or two-way ANOVA, respectively; p values <0.05 were considered statistically significant.

## Results

### Patient characteristics and basic anesthetic data

There were no significant differences in mean age, height or weight, or the ratio of men to women, between the groups. There were also no significant differences in mean nasopharyngeal temperature before surgery, duration of anesthesia, duration of surgery, bleeding, PONV and flurbiprofen dose among the groups. However the patients used epidural anesthesia as postoperative analgesia were more in HT(−) group than the other groups. Amount of fentanyl during surgery in the HT(+) group was significantly larger than that in the HT(−) group. Amount of Mg^2+^ during surgery was also higher in HT(−) group. The dose of fentanyl of iv-PCA was 25 μg/h as median value in all groups (Table 1).

**Table 1:** Participants’ demographic and clinical characteristics

	LT(+)	LT(−)	HT(+)	HT(−)
*Male/Total (n)	11/16	10/15	13/17	10/15
Age (yr)	64 [43, 75]	67 [50, 80]	61 [47, 81]	64 [37, 77]
Hight (cm)	161 [148, 173]	160 [140, 168]	167 [151, 174]	163 [142, 175]
Weight (kg)	59 [44, 84]	52 [45, 72]	60 [48, 84]	58 [40, 75]
Nasopharyngeal Temperature(°C)	36.3 [35.5, 36.6]	36.1 [35.6, 36.7]	36.2 [35.2, 36.7]	36.2 [35.8, 36.9]
Duration of anesthesia (min)	324 [228, 578]	331 [228, 508]	326 [197, 444]	301 [129, 655]
Duration of surgery (min)	282 [170, 539]	291 [184, 451]	292 [151, 396]	256 [93, 612]
Bleeding (g)	335 [53, 2260]	518 [95, 1417]	360 [85, 2155]	290 [10, 2027]
Amount of Mg^2+^ during surgery (mEq)	1.55 [0.0, 3.0]	1.6 [0.7, 7.8]	1.2^#^ [0.2, 3.3]	3.9 [0.7, 7.9]
Amount of fentanyl during surgery (μg)	500 [400, 700]	400 [75, 600]	600^#^ [300, 700]	400 [50, 600]
The dose of fentanyl of iv-PCA (μg/h)	25 [20, 30]	25 [20, 30]	25 [20, 30]	25 [16, 30]
*Administration Ratio of Flurbiprofen	15/16	10/15	15/17	9/15
*PONV	0/16	1/15	0/17	0/15
*The patients administrated Additional analgesics after surgery	2/16	3/15	6/17	5/15
*The case using iv-PCA	15/16	12/15	17/17^#^	7/15
*The case using epidural anesthesia	0/16	3/15	0/17^#^	7/15
Number	16	15	17	15

Data are presented as median [minimum, maximum]. One-way analysis of variance with Dunn’s multiple comparison test or Fisher’s exact test (*) with Holm-sidak test was used to compare each group with every other. Fisher’s exact test (*) was used to compare

*Abbreviation*: *LT(+)* low-dose remifentanil group treated with tramadol, *LT(−)* low-dose remifentanil not treated with tramadol, *HT(+)* high-dose remifentanil group treated with tramadol, *HT(−)* high-dose remifentanil group not treated with tramadol, *n.s.* not significant, *PONV* postoperative nausea and vomiting

^#^ *p <* 0.05 versus HT(−) group

### Operative methods

Operative methods in all groups were shown (Table 2).

**Table 2:** Number of Undertaken surgery in each group

	LT(+)	LT(−)	HT(+)	HT(−)	all
Distal Gastrectomy	7	8	7	7	29
Total Gastrectomy	6	5	6	3	20
Hepatectomy	1	1	3	1	6
Pancreaticoduodenectomy	1	1	0	2	4
Distal Pancreatectomy	1	0	0	0	1
Pancreaticogastrostomy	0	0	1	0	1
Subtotal Gastrectomy	0	0	0	1	1
Gastrojejunostomy	0	0	0	1	1
total	16	15	17	15	62

Operative methods undertaken in each group

### Post-anesthetic shivering

**Fig. 1:**
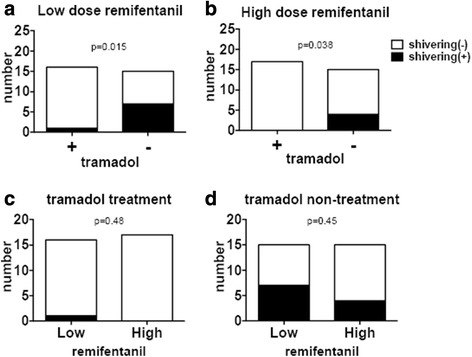
The effects of tramadol or remifentanil dose on the incidence of postoperative shivering: (**a**) the low-dose remifentanil group (*n* = 31) included 16 who were treated with tramadol treatment [LT(+)] and 15 who were not [LT(−)] and (**b**) the high-dose remifentanil group (*n* = 32) included 17 treated with tramadol [HT(+)] and 15 who were not [HT(−)]; (**c**) the tramadol treatment group (*n* = 33) included 17 administered high-dose remifentanil and 16 who were administered low-dose remifentanil, while (**d**) the group not administered tramadol group (*n* = 30) included 15 who received high-dose remifentanil and 15 who received low-dose remifentanil. The white portion represents those patients who did not shiver postoperatively, and the black portion those who experienced postoperative shivering. Fisher’s exact test was used for statistical analyses

In the low-dose remifentanil group, the incidence of PAS was significantly lower in the tramadol-treated group than the group who did not receive tramadol (one out of 16 (6.25%) compared with seven out of 15 (46.7%), respectively, *p* = 0.015). Similarly, the incidence of PAS in the high-dose remifentanil group was significantly lower in the tramadol-treated group than the group who did not receive tramadol (none out of 17 (0%) compared with four out of 15 (26.7%), respectively, *p* = 0.038). The dose of remifentanil did not appear to influence the incidence of PAS in those treated with tramadol (none out of 17 (0%) in the HT(+) group compared with one out of 16 (6.25%) in the LT(+) group; *p* = 0.48), or those not treated with tramadol (four out of 15 (26.7%) in the HT(−) group compared with seven out of 15 (46.7%) in the LT(−) group, *p* = 0.45; Fig. 1).

### Perioperative change in nasopharyngeal temperature

Mean nasopharyngeal temperature at the end of surgery was significantly higher in the HT(−) group than the HT(+) group (36.4 ± 0.4 °C compared with 35.6 ± 0.6 °C, respectively; *p =*0.008). Similarly, the perioperative temperature change; the temperature before surgery minus that after surgery; was significantly greater in the HT(+) than the HT(−) group (0.4 ± 0.5 °C compared with −0.2 ± 0.3 °C, respectively; *p =*0.040). There was no difference in mean nasopharyngeal temperature after surgery between the LT(+) and LT(−) groups (35.6 ± 0.9 °C compared with 36.1 ± 0.5 °C, respectively; *p =* 0.18). There was also no difference in perioperative temperature change in the LT(+) than the LT(−) group (0.6 ± 0.9 °C compared with 0.0 ± 0.5 °C, respectively; *p =* 0.06).

## Discussion

We found that tramadol significantly decreased the incidence of PAS after remifentanil anesthesia, regardless of the dose of remifentanil (Fig. 1a). Tramadol inhibits neuronal reuptake of norepinephrine and 5-hydroxytryptamine, and activates μ-opioid receptors, with minimal activity at κ- or σ-receptors [17]. Tramadol also reportedly inhibits the *N*-methyl-D-aspartic acid (NMDA) receptor at clinically relevant concentrations [18]. Pethidine is widely used to prevent PAS, while tramadol has also been reported to be effective [19]. Seifi et al. also reported that pethidine 0.5 mg/kg is as effective as tramadol 1 mg/kg for PAS during the first 15 min after injection, while tramadol is associated with less side effects [20]. Heid et al. reported that administration of 2 mg/kg tramadol reduced the incidence and extent of PAS after lumbar disc surgery under remifentanil-isoflurane general anesthesia [16]. Mohta et al. also reported that tramadol 2 mg/kg provided an effective combination of anti-PAS activity and analgesia without excessive sedation [8]. Moreover, Heidari et al. found that premedication with oral tramadol reduced the severity of PAS [9]. In preliminary study, we also originally administered tramadol 2 mg/kg, but PAS still occasionally occurred. However shivering hardly occurred after we administered tramadol 200 mg. Then we administered tramadol 200 mg at induction of anesthesia, equating to approximately 3 mg/kg, followed by 100 mg every 4 h. The interval of 4 h was estimated from the biological half-time of tramadol, which is in the region of 5 h when administered orally. Kose et al. have reported that ketamine was effective for PAS [21]. Ketamine is a competitive antagonist at the NMDA receptor, suggesting that the anti-shivering effect of tramadol might be mediated by the NMDA receptor.

In this study, mean nasopharyngeal temperatures were not markedly reduced in the non-tramadol treatment group (HT(−); from 36.3 ± 0.37 to 36.4 ± 0.4, LT(−); from 36.1 ± 0.29 to 36.1 ± 0.5). This reason is that non-shivering thermogenesis might be occurred. Additionally perioperative nasopharyngeal temperature fell significantly more in the HT(+) and LT(+) groups than the HT(−) and LT(−) groups. Tramadol may suppress the central nervous system temperature regulatory center causing dissipation of heat from the core to the periphery, while maintaining core temperature above the threshold for shivering. This is an interesting observation, as low core temperature at the time of completion of surgery is generally considered to pose a high risk for shivering.

We focused on shivering after remifentanil anesthesia. Ultiva® contains 15 mg glycine and hydrochloric acid. Glycine is required for the activation of the NMDA receptor [22]. Assuming that the NMDA receptor is involved in shivering, the PAS after remifentanil anesthesia might be a consequence of activation of NMDA receptors by the glycine contained in the drug preparation. Thus, tramadol might effectively suppress PAS by inhibiting the NMDA receptor. Short-acting opioids may cause acute opioid tolerance and hyperalgesia [23]; indeed Nakasuji et al. suggested that PAS was a consequence of opioid withdrawal caused by acute tolerance [7]. Wu et al. reported that NMDA receptor antagonists did not significantly reduce the incidence of PAS [24]; however, their cohort included patients undergoing septorhinoplasty or thyroidectomy, after which the incidence of PAS is recognized to be low.

**Fig. 2:**
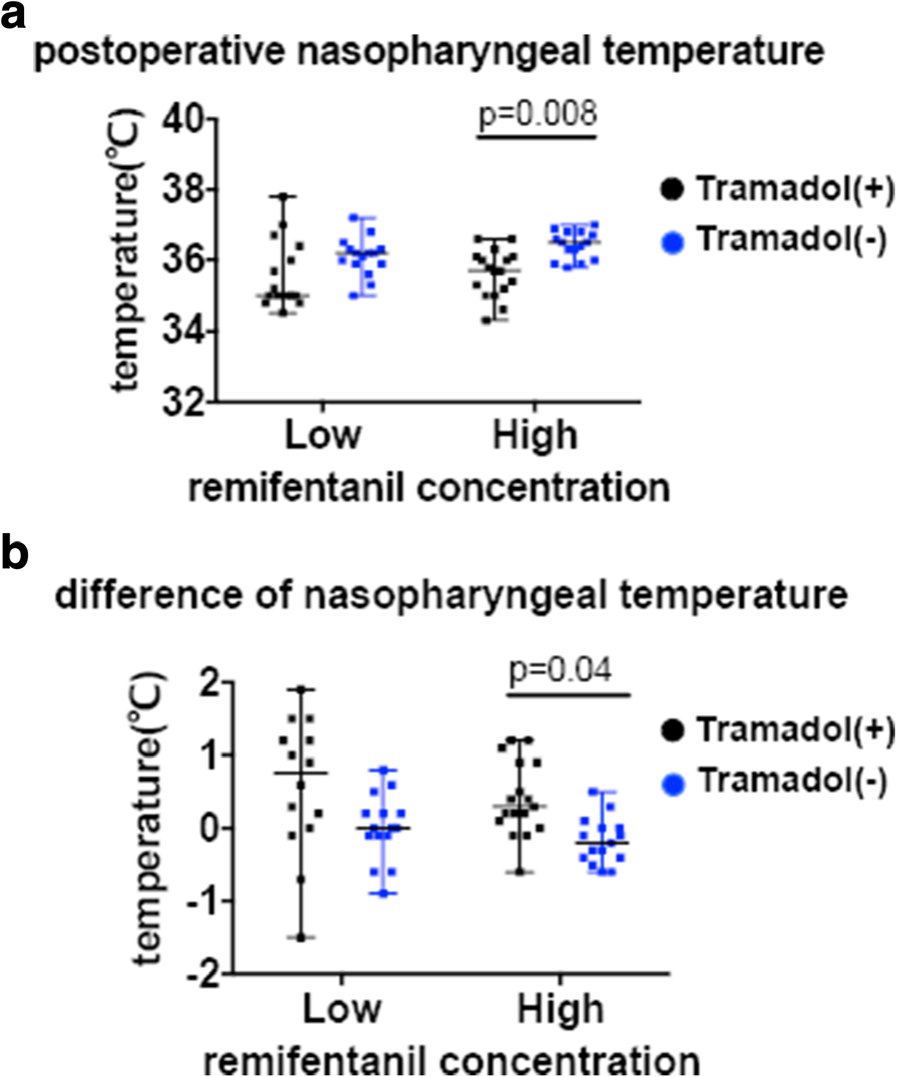
Postoperative nasopharyngeal temperature and perioperative nasopharyngeal temperature change: (**a**) nasopharyngeal temperature after surgery and (**b**) change from before surgery to after surgery in the high-dose remifentanil group. Ordinary two-way analysis of variance (ANOVA) was used for statistical analyses. Tukey’s multiple comparison *post hoc* tests were performed for two-way ANOVA. The upper, middle and lower bars indicate maximum, median and minimum value each

In this study, we obtained interesting results regarding nasopharyngeal temperature. Administration of a high concentration of remifentanil caused a greater but non-significant increase of nasopharyngeal temperature compared with the low concentration (Fig. 2a, HT(−) vs. LT(−), *p =* 0.069). Moreover, the use of tramadol from the start of Ultiva® administration decreased nasopharyngeal temperature in the high concentration of remifentanil group (Fig. 2a). Given these results, we propose four possible explanations. First, administration of Ultiva® might increase core temperature, while shivering might not occur during administration. Second, the hyperthermic effect of Ultiva® might be related to stimulation of the NMDA receptor by glycine in Ultiva®. Third, shivering might not occur during Ultiva® administration because remifentanil in Ultiva® or other anesthetics administered simultaneously suppressed shivering. Fourth, shivering after remifentanil anesthesia might be a result of the sudden decrease of the ability of remifentanil to suppress shivering. We believe there may be complex thermoregulatory mechanisms via the NMDA receptor and opioid receptor (ex-vivo), which may account for the occurrence of shivering (in-vivo).

In this study, while there were no differences in nasopharyngeal temperature between the LT(−) group and LT(+) group at the end of surgery, it was significantly higher in the HT(−) group compared with the HT(+) group. The reason might be that the low-dose glycine (low-dose Ultiva®) did not sufficiently activate NMDA receptors in the low remifentanil concentration groups LT(−) group. The lowering effect on body temperature via suppression of the thermoregulatory center by tramadol might be therefore too small to exceed the significance threshold in the LT(+) group. By contrast, the high-dose glycine might sufficiently activate NMDA receptors such that a strong hyperthermic response occurred in the HT(−) group, and the body temperature-lowering effect of tramadol might exceed the threshold in the HT(+) group. Further research is required to fully understand the relationship between PAS and the NMDA receptor.

We found no relationship between the dose of remifentanil and the incidence of PAS. Nakasuji et al. reported that the incidence of PAS was higher after high-dose remifentanil (0.25 μg/kg/min) than a low-dose regime (0.1 μg/kg/min) [7]. We defined low dose as 0.1–0.25 μg/kg/min and high dose as 1–1.5 μg/kg/min. Differences between our findings and those of past studies might be a consequence of different definitions.

## Limitations

This study has some limitations. First, its design was retrospective and patients were not randomized. In HT(−) group, the number of patients used epidural anesthesia was larger than another group (LT(−): 3 out of 15, HT(−): 7 out of 15, LT(+): 0 out of 16, HT(+) 0 out of 17). Moreover, the amount of fentanyl administered during surgery and the estimated effect site concentration of fentanyl at end of surgery were not standardized. In this study, the incidence of PAS was significantly lower in the patients who used epidural anesthesia than the patients who used iv-PCA (none out of 7 (0%) compared with four out of 7 (57.1%), respectively; *p =* 0.018, not shown) in HT(−) group. Therefore, epidural anesthesia may be superior to fentanyl administration in PAS. Second, we studied only those undergoing upper abdominal surgery and the duration of surgery varied widely, likely resulting in wide variation in surgical stress that might have influenced the incidence of PAS. Third, we assessed only whether shivering occurred or not. Assessing the extent and duration of shivering might further illuminate the effect of tramadol. Ideally, a trained observer should record shivering in a reliable and standardized manner, but we could not. Fourth, we did not measure peripheral temperature; the central-peripheral temperature difference might have been influenced by tramadol. Finally, we did not standardize flurbiprofen or fentanyl administration. Although flurbiprofen did not influence the incidence of PAS in our study, there was possibility that non-steroidal anti-inflammatory drugs might have effects on the distribution of body heat from the core to the periphery. The small sample size per group (15–17 patients) might not provide effects of flubiprofen on shivering.

Further prospective studies are needed to examine the effects of tramadol on PAS.

## Conclusion

Post-anesthetic shivering appeared to occur independently of remifentanil concentration. Administration of tramadol significantly reduced the incidence of PAS independently of remifentanil concentration. Tramadol may be a useful means of preventing PAS.

## Abbreviations

HT (−): High-dose remifentanil (1–1.5 μg/kg/min) without tramadol group
HT (+): High-dose remifentanil (1–1.5 μg/kg/min) with tramadol group
iv-PCA: Intravenous patient controlled analgesia
LT(−): low-dose remifentanil (0.15–0.25 μg/kg/min) without tramadol
LT(+): Low-dose remifentanil (0.15–0.25 μg/kg/min) with tramadol group
NMDA: *N*-methyl-D-aspartic acid
PAS: Post-anesthetic shivering
PONV: Postoperative nausea and vomiting
